# Supplementary material for: MALTectomy and psoriasis risk in women: A prospective study in the French E3N prospective cohort
Source: PLoS One. 2024 Nov 18;19(11):e0310891. doi: 10.1371/journal.pone.0310891 (PMC11573222; doi:10.1371/journal.pone.0310891)
Supplement: S1 File — (DOCX) [file pone.0310891.s001.docx]

**MALTectomy and psoriasis risk in women: a prospective study in the French E3N prospective cohort.**

Marco Conte, Agnes Fournier, Joseph A Rothwell, Marie-Christine Boutron-Ruault, Laura Baglietto, Marco Fornili, Emilie Sbidian, Gianluca Severi

**Supplementary Tables**

**Table 1.** List of drugs that can be indicated for the treatment of psoriasis extracted from MGEN drug reimbursement database.

**Table 2.** Characteristics of study participants according to exclusion status in the overall psoriasis population compared to the entire E3N population (n = 98 995), E3N Cohort, France, 1990–2018.

**Table 3.** Characteristics of study participants according to appendectomy status at the end of follow-up (n = 90 119), E3N Cohort, France, 1990–2018.

**Table 4.** Characteristics of study participants according to adenoidectomy status at the end of follow-up (n = 90 119), E3N Cohort, France, 1990–2018.

**Table 5.** Characteristics of study participants according to tonsillectomy status at the end of follow-up (n = 90 119), E3N Cohort, France, 1990–2018.

**Table 6.** Characteristics of study participants according to moderate-to-severe status at the end of follow-up (n = 78 269), E3N Cohort, France, 1990–2018.

**Table 7.** Hazard ratio for the associations between age at appendectomy and psoriasis risk (n = 90 119), Etude epidémiologique auprès de femmes de l’Education Nationale Cohort, France, 1990–2018

**Table 8.** Hazard ratio for the associations between age at adeinoidectomy and psoriasis risk (n = 90 119), Etude epidémiologique auprès de femmes de l’Education Nationale Cohort, France, 1990–2018

**Table 9.** Hazard ratio for the associations between age at tonsillectomy and psoriasis risk (n = 90 119), Etude epidémiologique auprès de femmes de l’Education Nationale Cohort, France, 1990–2018

**Table 10.** Hazard ratio for the associations between appendectomy as time-varying variable and psoriasis risk (n = 90 119), Etude epidémiologique auprès de femmes de l’Education Nationale Cohort, France, 1990–2018

**Table 11.** Hazard Ratios of psoriasis risk according to MALTectomy history further adjusted for alcohol consumption, (n = 70 350), Etude epidémiologique auprès de femmes de l’Education Nationale Cohort, France, 1990–2018

**Table 1. List of drugs that can be indicated for the treatment of psoriasis extracted from MGEN drug reimbursement database.**

| **Topical drugs derived from vitamin D** | Calcipotriol  Calcipotriol in association with betamethasone  Calcitriol  Tacalcitol  Tazarotene |
| --- | --- |
| **Topical steroids** | Betamethasone  Clobetasol  Desonide  Diflucortolone  Difluprednate  Fluticasone  Hydrocortisone Aceponate  Hydrocortisone Butyrate |
| **Psoralens** | Methoxsalene |
| **Non-biologic immunosuppressants** | Acitretine  Ciclosporine  Methotrexate |
| **Biologic immunosuppressants** | Adalinumab  Apremilast  Etanercept  Ixekizumab  Secukinumab  Ustekinumab |

Abbreviations: MGEN- Mutuelle générale de l'Éducation nationale

**Table 2.** **Characteristics of study participants according to exclusion status in the overall psoriasis population compared to the entire E3N population (n = 98 995), E3N Cohort, France, 1990–2018.**

|  | **Exclusion status** | |
| --- | --- | --- |
|  | **Excluded population**  **(n= 8 876)** | **No Appendectomy**  **(n= 90 119)** |
| **Psoriasis** |  |  |
| No | 5 150 (58.0%) | 87 686 (97.3%) |
| Yes | 3 726 (42.0%) | 2 433 (2.7%) |
| **Appendectomy** | | |
| No | 3 896 (43.9%) | 55 731 (61.8%) |
| Yes | 2 726 (30.7%) | 34 388 (38.2%) |
| Missing | 2 254 (25.4%) | 0 (0%) |
| **Adenoidectomy** | | |
| No | 4 875 (54.9%) | 65 315 (72.5%) |
| Yes | 1 970 (22.5%) | 24 804 (27.5%) |
| Missing | 2 031 (22.9) | 0 (0%) |
| **Tonsillectomy** | | |
| No | 4 568 (51.5%) | 61 720 (68.5%) |
| Yes | 2 294 (25.8%) | 28 399 (31.5%) |
| Missing | 2 014 (22.7%) | 0 (0%) |
| **Year of birth** | | |
| >1945 | 3 179 (35.8%) | 30 359 (33.7%) |
| 1940-1945 | 2 146 (24.2%) | 21 780 (24.2%) |
| 1935-1940 | 1 668 (18.8%) | 17 768 (19.7%) |
| 1930-1935 | 1 137 (12.8%) | 12 060 (13.4%) |
| <1930 | 746 (8.4%) | 8 152 (9.0%) |
| **BMI at baseline** | | |
| BMI<25 | 6 935 (78.1%) | 73 861 (82.0%) |
| 25<BMI<30 | 1 515 (17.1%) | 13 417 (14.9%) |
| BMI>30 | 426 (4.8%) | 2 841 (3.2%) |
| **Smoking status at baseline** | | |
| Never | 4 911 (55.3%) | 48 735 (54.1%) |
| Current | 1 497 (16.9%) | 13 383 (14.9%) |
| Former | 2 468 (27.8%) | 28 001 (31.1%) |
| **Marital status at baseline** | | |
| Unmarried | 1688 (19.0%) | 15 917 (17.7%) |
| Married | 7 188 (81.0%) | 74 202 (82.3%) |
| **Education level** | | |
| Undergraduate or less | 633 (7.1%) | 3 097 (3.4%) |
| Graduate | 1 377 (15.5%) | 12 056 (13.4%) |
| Postgraduate or more | 6 866 (77.4%) | 74 966 (83.2%) |
| **Age at menarche** | | |
| 12-15 years old | 6 802 (76.6) | 68 244 (75.7%) |
| <12 years old | 1 721 (19.4%) | 18 337 (20.3%) |
| >15 years old | 353 (4.0%) | 3 538 (3.9%) |
| **Menopause status at baseline** | | |
| Premenopausal | 4 858 (54.7%) | 46 142 (51.2%) |
| Postmenopausal | 4 018 (45.3%) | 43 977 (48.8%) |
| **Nulliparous status** | | |
| No | 7 350 (82.9%) | 79 568 (88.3%) |
| Yes | 1 516 (17.1%) | 10 551 (11.7%) |
| **Ever use of oral contraceptives at baseline** | | |
| No | 4 360 (49.1%) | 40 450 (44.9%) |
| Yes | 4 516 (50.9%) | 49 669 (55.1%) |

**Table 3.** **Characteristics of study participants according to appendectomy status at the end of follow-up (n = 90 119), E3N Cohort, France, 1990–2018.**

|  | **Appendectomy status** | |
| --- | --- | --- |
|  | **Appendectomy**  **(n= 34 388)** | **No Appendectomy**  **(n= 55 731)** |
| **Psoriasis** |  |  |
| No | 33 372 (97.0%) | 54 314 (97.5%) |
| Yes | 1 016 (3.0%) | 1 417 (2.5%) |
| **Adenoidectomy** | | |
| No | 23 433 (68.1%) | 41 882 (75.2%) |
| Yes | 10 955 (31.9%) | 13 849 (24.8%) |
| **Tonsillectomy** | | |
| No | 21 746 (63.2%) | 39 974 (71.7%) |
| Yes | 12 642 (36.8%) | 15 757 (28.3%) |
| **Year of birth** | | |
| >1945 | 11 184 (32.5%) | 19 175 (34.4%) |
| 1940-1945 | 8 572 (24.9%) | 13 208 (23.7%) |
| 1935-1940 | 7 069 (20.6%) | 10 699 (19.2%) |
| 1930-1935 | 4 563 (13.3%) | 7 497 (13.5%) |
| <1930 | 3 000 (8.7%) | 5 152 (9.2%) |
| **BMI at baseline** | | |
| BMI<25 | 27 588 (80.2%) | 46 273 (83.0%) |
| 25<BMI<30 | 5 538 (16.1%) | 7 879 (14.1%) |
| BMI>30 | 1 262 (3.7%) | 1 579 (2.8%) |
| **Smoking status at baseline** | | |
| Never | 18 032 (52.4%) | 30 703 (55.1%) |
| Current | 5 297 (15.4%) | 8 086 (14.5%) |
| Former | 11 059 (32.2%) | 16 942 (30.4%) |
| **Marital status at baseline** | | |
| Unmarried | 6 038 (17.6%) | 9 879 (17.7%) |
| Married | 28 350 (82.4%) | 45 852 (82.3%) |
| **Education level** | | |
| Undergraduate or less | 1 160 (3.4%) | 1 937 (3.5%) |
| Graduate | 5 287 (15.4%) | 6 769 (12.1%) |
| Postgraduate or more | 27 941 (81.3%) | 47 025 (84.4%) |
| **Age at menarche** | | |
| 12-15 years old | 25 674 (74.7%) | 42 570 (76.4%) |
| <12 years old | 7 374 (21.4%) | 10 963 (19.7%) |
| >15 years old | 1 340 (3.9%) | 2 198 (3.9%) |
| **Menopause status at baseline** | | |
| Premenopausal | 17 107 (49.7%) | 29 035 (52.1%) |
| Postmenopausal | 17 281 (50.3%) | 26 696 (47.9%) |
| **Nulliparous status** | | |
| No | 30 610 (89.0%) | 48 958 (87.8%) |
| Yes | 3 778 (11.0%) | 6 773 (12.2%) |
| **Ever use of oral contraceptives at baseline** | | |
| No | 15 485 (45.0%) | 24 965 (44.8%) |
| Yes | 18 903 (55.0%) | 30 766 (55.2%) |

**Table 4. Characteristics of study participants according to adenoidectomy status at the end of follow-up (n = 90 119), E3N Cohort, France, 1990–2018.**

|  | **Adenoidectomy status** | |
| --- | --- | --- |
|  | **Adenoidectomy**  **(n= 28 399)** | **No Adenoidectomy**  **(n= 61 720)** |
| **Psoriasis** |  |  |
| No | 24 101 (97.2%) | 63 585 (97.4%) |
| Yes | 703 (2.8%) | 1 730 (2.6%) |
| **Appendectomy** |  |  |
| No | 10 955 (44.2%) | 23 433 (35.9%) |
| Yes | 13 849 (55.8%) | 41 882 (64.1%) |
| **Tonsillectomy** |  |  |
| No | 6 319 (25.5%) | 55 401 (84.8%) |
| Yes | 18 485 (74.5%) | 9 914 (15.2%) |
| **Year of birth** |  |  |
| >1945 | 8 845 (35.7%) | 21 514 (32.9%) |
| 1940-1945 | 6 061 (24.4%) | 15 719 (24.1%) |
| 1935-1940 | 4 974 (20.1%) | 12 794 (19.6%) |
| 1930-1935 | 2 972 (12.0%) | 9 088 (13.9%) |
| <1930 | 1 952 (7.9%) | 6 200 (9.5%) |
| **BMI at baseline** |  |  |
| BMI<25 | 20 338 (82.0%) | 53 523 (81.9%) |
| 25<BMI<30 | 3 661 (14.8%) | 9 756 (14.9%) |
| BMI>30 | 805 (3.2%) | 2 036 (3.1%) |
| **Smoking status at baseline** |  |  |
| Never | 12 848 (51.8%) | 35 887 (54.9%) |
| Current | 3 754 (15.1%) | 9 629 (14.7%) |
| Former | 8 202 (33.1%) | 19 799 (30.3%) |
| **Marital status at baseline** |  |  |
| Unmarried | 4 584 (18.5%) | 11 333 (17.4%) |
| Married | 20 220 (81.5%) | 53 982 (82.6%) |
| **Education level** |  |  |
| Undergraduate or less | 805 (3.2%) | 2 292 (3.5%) |
| Graduate | 2 621 (10.6%) | 9 435 (14.4%) |
| Postgraduate or more | 21 378 (86.2%) | 53 588 (82.0%) |
| **Age at menarche** |  |  |
| 12-15 years old | 18 509 (74.6%) | 49 735 (76.1%) |
| <12 years old | 5 425 (21.9%) | 12 912 (19.8%) |
| >15 years old | 870 (3.5%) | 2 668 (4.1%) |
| **Menopause status at baseline** |  |  |
| Premenopausal | 13 213 (53.3%) | 32 929 (50.4%) |
| Postmenopausal | 11 591 (46.7%) | 32 386 (49.6%) |
| **Nulliparous status** |  |  |
| No | 21 727 (87.6%) | 57 841 (88.6%) |
| Yes | 3 077 (12.4%) | 7 474 (11.4%) |
| **Ever use of oral contraceptives at baseline** |  |  |
| No | 10 614 (42.8%) | 29 836 (45.7%) |
| Yes | 14 190 (57.2%) | 35 479 (54.3%) |

**Table 5. Characteristics of study participants according to tonsillectomy status at the end of follow-up (n = 90 119), E3N Cohort, France, 1990–2018.**

|  | **Tonsillectomy status** | |
| --- | --- | --- |
|  | **Tonsillectomy**  **(n= 28 399)** | **No Tonsillectomy**  **(n= 61 720)** |
| **Psoriasis** |  |  |
| No | 27 619 (97.3%) | 60 067 (97.3%) |
| Yes | 780 (2.7%) | 1 653 (2.7%) |
| **Appendectomy** |  |  |
| No | 12 642 (44.5%) | 21 746 (35.2%) |
| Yes | 15 757 (55.5%) | 39 974 (64.8%) |
| **Adenoidectomy** |  |  |
| No | 18 485 (65.1%) | 6 319 (10.2%) |
| Yes | 9 914 (34.9%) | 55 401 (89.8%) |
| **Year of birth** |  |  |
| >1945 | 10 118 (35.6%) | 20 241 (32.8%) |
| 1940-1945 | 7 081 (24.9%) | 14 699 (23.8%) |
| 1935-1940 | 5 598 (19.7%) | 12 170 (19.7%) |
| 1930-1935 | 3 376 (11.9%) | 8 684 (14.1%) |
| <1930 | 2 226 (7.8%) | 5 926 (9.6%) |
| **BMI at baseline** |  |  |
| BMI<25 | 23 024 (81.1%) | 50 837 (82.4%) |
| 25<BMI<30 | 4 388 (15.5%) | 9 029 (14.6%) |
| BMI>30 | 987 (3.5%) | 1 854 (3.0%) |
| **Smoking status at baseline** |  |  |
| Never | 14 792 (52.1%) | 33 943 (55.0%) |
| Current | 4 333 (15.3%) | 9 050 (14.7%) |
| Former | 9 274 (32.7%) | 18 727 (30.3%) |
| **Marital status at baseline** |  |  |
| Unmarried | 5 201 (18.3%) | 10 716 (17.4%) |
| Married | 23 198 (81.7%) | 51 004 (82.6%) |
| **Education level** |  |  |
| Undergraduate or less | 960 (3.4%) | 2 137 (3.5%) |
| Graduate | 3 540 (12.5%) | 8 516 (13.8%) |
| Postgraduate or more | 23 899 (84.2%) | 51 067 (82.7%) |
| **Age at menarche** |  |  |
| 12-15 years old | 21 304 (75.0%) | 46 940 (76.1%) |
| <12 years old | 6 072 (21.4%) | 12 265 (19.9%) |
| >15 years old | 1 023 (3.6%) | 2 515 (4.1%) |
| **Menopause status at baseline** |  |  |
| Premenopausal | 15 126 (53.3%) | 31 016 (50.3%) |
| Postmenopausal | 13 273 (46.7%) | 30 704 (49.7%) |
| **Nulliparous status** |  |  |
| No | 25 010 (88.1%) | 54 558 (88.4%) |
| Yes | 3 389 (11.9%) | 7 162 (11.6%) |
| **Ever use of oral contraceptives at baseline** |  |  |
| No | 12 223 (43.0%) | 28 227 (45.7%) |
| Yes | 16 176 (57.0%) | 33 493 (54.3%) |

**Table 6. Characteristics of study participants according to moderate-to-severe status at the end of follow-up (n = 78 269), E3N Cohort, France, 1990–2018.**

|  | **Moderate-to-severe status** | |
| --- | --- | --- |
|  | **Moderate-to-severe psoriasis cases**  **(n= 120)** | **Not moderate-to-severe psoriasis cases**  **(n= 78 149)** |
| **Appendectomy** |  |  |
| No | 64 (53.3%) | 48 445 (62.0%) |
| Yes | 56 (46.7%) | 29 704 (38.0%) |
| **Adenoidectomy** |  |  |
| No | 84 (70.0%) | 56 614 (72.4%) |
| Yes | 36 (30.0%) | 21 535 (27.6%) |
| **Tonsillectomy** |  |  |
| No | 83 (69.2%) | 53 547 (68.5%) |
| Yes | 37 (30.8%) | 24 602 (31.5%) |
| **Year of birth** |  |  |
| >1945 | 56 (46.7%) | 27 064 (34.6%) |
| 1940-1945 | 23 (19.2%) | 19 331 (24.7%) |
| 1935-1940 | 24 (20.0%) | 15 577 (19.9%) |
| 1930-1935 | 11 (9.2%) | 10 064 (12.9%) |
| <1930 | 6 (5.0%) | 6 113 (7.8%) |
| **BMI at baseline** |  |  |
| BMI<25 | 93 (77.5%) | 64 897 (83.0%) |
| 25<BMI<30 | 24 (20.0%) | 11 045 (14.1%) |
| BMI>30 | 3 (2.5%) | 2 207 (2.8%) |
| **Smoking status at baseline** |  |  |
| Never | 52 (43.3%) | 42 135 (53.9%) |
| Current | 28 (23.3%) | 11 170 (14.3%) |
| Former | 40 (33.3%) | 24 844 (31.8%) |
| **Marital status at baseline** |  |  |
| Unmarried | 29 (24.2%) | 13 189 (16.9%) |
| Married | 91 (75.8%) | 64 960 (83.1%) |
| **Education level** |  |  |
| Undergraduate or less | 6 (5.0%) | 2 666 (3.4%) |
| Graduate | 11 (9.2%) | 9 893 (12.7%) |
| Postgraduate or more | 103 (85.8%) | 65 590 (83.9%) |
| **Age at menarche** |  |  |
| 12-15 years old | 86 (71.7%) | 59 348 (75.9%) |
| <12 years old | 30 (25.0%) | 15 781 (20.2%) |
| >15 years old | 4 (3.3%) | 3 020 (3.9%) |
| **Menopause status at baseline** |  |  |
| Premenopausal | 74 (61.7%) | 41 293 (52.8%) |
| Postmenopausal | 46 (38.3%) | 36 856 (47.2%) |
| **Nulliparous status** |  |  |
| No | 102 (85.0%) | 69 280 (88.7%) |
| Yes | 18 (15.0%) | 8 869 (11.3%) |
| **Ever use of oral contraceptives at baseline** |  |  |
| No | 40 (33.3%) | 34 281 (43.9%) |
| Yes | 80 (66.7%) | 43 868 (56.1%) |

**Table 7. Hazard ratio for the associations between age at appendectomy and psoriasis risk (n = 90 119), Etude epidémiologique auprès de femmes de l’Education Nationale Cohort, France, 1990–2018.**

| Appendectomy  Time-intervals (years) | **No psoriasis cases**  **(N=87 686)** | **Psoriasis cases**  **(N=2 433)** | **Univariate model^a^** | **p-value** |
| --- | --- | --- | --- | --- |
|  |  |  | HR  (95% CI) |  |
| No appendectomy | 54 314 | 1 417 | 1.00  (Reference) |  |
| <10 | 5 583 | 200 | 1.33  (0.92-1.94) | 0.12 |
| 11-14 | 5 320 | 174 | 1.23  (0.84-1.80) | 0.27 |
| 15-19 | 7 327 | 211 | 1.11  (0.77-1.61) | 0.56 |
| 20-29 | 8 918 | 269 | 1.18  (0.83-1.27) | 0.36 |
| 30-39 | 3 582 | 94 | 1.05  (0.70-1.57) | 0.78 |
| >40 | 1 377 | 36 | 1.12  (0.69-1.80) | 0.63 |
| Appendectomy cases without diagnostic date | 1 265 | 32 | Not  computable | Not  computable |

1. Model adjusted for: appendectomy status, age at baseline.

**Table 8. Hazard ratio for the associations between age at adeinoidectomy and psoriasis risk (n = 90 119), Etude epidémiologique auprès de femmes de l’Education Nationale Cohort, France, 1990–2018.**

| Adenoidectomy  Time-intervals (years) | **No psoriasis cases**  **(N=87 686)** | **Psoriasis cases**  **(N=2 433)** | **Univariate model^a^** | **p-value** |
| --- | --- | --- | --- | --- |
|  |  |  | HR  (95% CI) |  |
| No adeinodectomy | 65 520 | 1 784 | 1.00  (Reference) |  |
| <10 | 19 790 | 587 | 1.02  (0.77-1.35) | 0.85 |
| 11-14 | 1 823 | 51 | 1.01  (0.69-1.48) | 0.93 |
| 15-19 | 329 | 6 | 0.65  (0.28-1.53) | 0.33 |
| 20-29 | 151 | 3 | 0.75  (0.23-2.42) | 0.64 |
| 30-39 | 40 | 1 | 0.95  (0.13-6.91) | 0.96 |
| >40 | 9 | 0 | Not  computable | Not  computable |
| Adeinodectomy cases without diagnostic date | 24 | 1 | 1.57  (0.21-11.37) | 0.65 |

1. Model adjusted for: adenoidectomy status, age at baseline.

**Table 9. Hazard ratio for the associations between age at tonsillectomy and psoriasis risk (n = 90 119), Etude epidémiologique auprès de femmes de l’Education Nationale Cohort, France, 1990–2018.**

| Tonsillectomy  Time-intervals (years) | **No psoriasis cases**  **(N=87 686)** | **Psoriasis cases**  **(N=2 433)** | **Univariate model^a^** | **p-value** |
| --- | --- | --- | --- | --- |
|  |  |  | HR  (95% CI) |  |
| No tonsillectomy | 61 523 | 1 689 | 1.00  (Reference) |  |
| <10 | 18 718 | 542 | 1.09  (0.78-1.53) | 0.60 |
| 11-14 | 2 349 | 65 | 1.08  (0.71-1.62) | 0.70 |
| 15-19 | 1 312 | 40 | 1.18  (0.75-1.85) | 0.46 |
| 20-29 | 2 753 | 76 | 1.07  (0.72-1.59) | 0.71 |
| 30-39 | 822 | 18 | 0.87  (0.49-1.53) | 0.63 |
| >40 | 168 | 3 | 0.76  (0.23-2.47) | 0.65 |
| Tonsillectomy cases without diagnostic date | 41 | 0 | Not  computable | Not  computable |

1. Model adjusted for: tonsillectomy status, age at baseline.

**Table 10. Hazard ratio for the associations between appendectomy as time-varying variable and psoriasis risk (n = 90 119), Etude epidémiologique auprès de femmes de l’Education Nationale Cohort, France, 1990–2018.**

| **Exposure** | **No psoriasis cases**  **(N=87 686)** | **Psoriasis cases**  **(N=2 433)** | **Hazard Ratio (95% CI)** | | | |
| --- | --- | --- | --- | --- | --- | --- |
|  |  |  | **Univariate model^a^** | **p-value** | **Multivariable model^b^** | **p-value** |
| Appendectomy |  |  |  |  |  |  |
| No | 54 352 | 1410 | 1.00  [Reference] |  | 1.00  [Reference] |  |
| Yes | 33 334 | 1023 | **1.16**  **(1.07-1.26)** | 0.002 | **1.13**  **(1.04-1.23)** | 0.002 |

1. Univariate model was age adjusted
2. Multivariate model was adjusted for BMI, smoking status, education level, marital status at baseline, hypertension, depression, diabetes, age at menarche, menopause status, nulliparous status, ever use of contraceptive pill.

**Table 11. Hazard Ratios of psoriasis risk according to MALTectomy history further adjusted for alcohol consumption, (n = 70 350), Etude epidémiologique auprès de femmes de l’Education Nationale Cohort, France, 1990–2018.**

| **Exposure** | **No psoriasis cases**  **(N=68 340)** | **Psoriasis cases**  **(N=2 010)** | **Hazard Ratio (95% CI)** | |
| --- | --- | --- | --- | --- |
|  |  |  | **Multivariable model^b^** | **p-value** |
| Appendectomy |  |  |  |  |
| No | 42 771 | 1 177 | 1.00  [Reference] |  |
| Yes | 25 569 | 833 | **1.16**  **(1.06-1.27)** | <0.001 |
| Adenoidectomy |  |  |  |  |
| No | 49 397 | 1 413 | 1.00  [Reference] |  |
| Yes | 18 943 | 597 | 1.07  (0.97-1.18) | 0.15 |
| Tonsillectomy |  |  |  |  |
| No | 46 754 | 1 354 | 1.00  [Reference] |  |
| Yes | 21 586 | 656 | 1.01  (0.92-1.11) | 0.71 |
